# Metabolic markers in Ossabaw pigs fed high fat diets enriched in regular or low α-linolenic acid soy oil

**DOI:** 10.1186/1743-7075-10-27

**Published:** 2013-03-14

**Authors:** Ramesh B Potu, Hang Lu, Olayiwola Adeola, Kolapo M Ajuwon

**Affiliations:** 1 Department of Animal Sciences, Purdue University, West Lafayette, IN 47907, USA

**Keywords:** Linolenic, Soy bean oil, Pigs, Obesity, Metabolic syndrome

## Abstract

**Background:**

Soy oil is a major vegetable oil consumed in the US. A recently developed soybean variety produces oil with a lower concentration of α-linolenic acid, hence a higher (n-6)/(n-3) ratio, than regular soy oil. The study was conducted to determine the metabolic impact of the low α-linolenic acid containing soy oil.

**Methods:**

Ossabaw pigs were fed diets supplemented with either 13% regular soybean oil (SBO), or 13% of the low α-linolenic soybean oil (LLO) or a control diet (CON) without extra oil supplementation, for 8 weeks.

**Results:**

Serum and adipose tissue α-linolenic acid concentration was higher in pigs fed the SBO diet than those on the CON and LLO diets. In the serum, the concentration of saturated fatty acids (SFA) was lower in the LLO group than in CON and SBO groups polyunsaturated fatty acid (PUFA) concentration was higher in the LLO group compared to CON and SBO groups. Glucose, insulin, triglycerides and LDL-cholesterol were higher in pigs fed the SBO diet than those fed the CON and LLO diets. HDL-cholesterol was lower in pigs on the SBO diet than those on the CON and LLO diets. Pigs fed SBO and LLO diets had lower CRP concentration than those on the CON diet. Adipose tissue expression of Interleukin 6 (IL-6) was higher in the SBO and LLO diets than the CON. Expression of ECM genes, COLVIA and fibronectin, was significantly reduced in the SBO diet relative to the CON and LLO diets whereas expression of inflammation-related genes, cluster of differentiation 68 (CD68) and monocyte chemoattractant protein 1 (MCP-1), was not different across treatments.

**Conclusions:**

Results suggest that lowering the content of α-linolenic acid in the context of a high fat diet could lead to mitigation of development of hyperinsulinemia and dyslipidemia without significant effects on adipose tissue inflammation.

## Background

Obesity is a state of chronic low-grade inflammation that is associated with insulin resistance, hyperlipidemia and cardiometabolic diseases [1,2]. Several studies have linked consumption of diets high in oils to an increased incidence of obesity [3-5]. In contrast, consumption of diets rich in unsaturated fatty acids such as soy oil, with its high content of linoleic and α-linolenic acids, is beneficial in reducing inflammation and serum lipid concentrations [6,7]. Consumption of polyunsaturated fatty acids is also associated with reduced cardiovascular disease risk [8]. Soy oil is the vegetable oil consumed in the largest amounts in American diets [9]. However, α-linolenic acid is rapidly degraded during processing and in storage due to its highly unsaturated structure. It oxidizes twice as quickly as linoleic acid [C18:2(n-6)] under stable conditions [10]. Therefore, a low α-linolenic acid soy oil was developed to improve the shelf life of the oil. The low α-linolenic acid oil thus eliminates the requirement for hydrogenation of soy oil due to the overall reduction in the content of unsaturated bonds. The low α-linolenic oil contains less than 3% α-linolenic acid versus the 7% in conventional soy oil [11]. Due to the alteration in the fatty acid composition of the low α-linolenic acid soy oil, it is important to study the effect of its consumption on major metabolic markers with respect to lipid metabolism, insulin sensitivity and inflammatory status. Therefore, the main objective of this work was to compare the metabolic and inflammatory impact of consumption of low α-linolenic soy oil vs. regular soy oil.

## Methods

### Animals and diets

The Purdue Animal Care and Use Committee (PACUC) approved all procedures on care and use of pigs described in this study. Twelve female Ossabaw pigs at 3 months of age were divided, four per group, into the three dietary treatments; Control diet (CON) with no extra oil addition, a high fat diet with 13% regular soybean oil diet (SBO) (from a local retail store), and a high fat diet with 13% low α-linolenic soybean oil diet (LLO) (Zeeland food services, Zeeland, MI). The initial weights of the pigs were 13.9, 13.7 and 14.2 kg for the CON, SBO and LLO treatments, respectively. The nutrient composition of experimental diets is presented in Table 1. The fatty acid composition of the regular soy oil and the low α-linolenic oil are also presented in Table 2. The fatty acid composition of diets is presented in Table 3. Pigs were penned individually at the Purdue small animal housing facility and were fed ad libitum for 8 wks. The total fat content of the soy oil diets (SBO and LLO) was increased to 19.7% by the addition of 13% of respective soy oils to a basal diet (5L80, Lab Diet, St. Louis, MO). The soy oil diets also contained 2% cholesterol and 0.67% sodium cholate by weight. Pigs were killed at the end of the study with intramuscular injection of atropine, tiletamine-zolazepam, and xylazine followed by pneumothorax and cardiectomy.

**Table 1:** Analyzed diet composition

**Ingredient (%)**	**Treatment**^**1**^		
	**CON**	**SBO**	**LLO**
Composition^*^			
Carbohydrates	64.5	45.9	45.9
Fat^2^	3.9	19.7	19.7
Protein	15.4	15.8	15.8
Cholesterol	0	2	2
Sodium Cholate	0	0.67	0.67
Corn Syrup	0	5	5
Minerals & Vitamins	6.2	6.2	6.2
**Amount of energy supplied**			
Carbohydrates (%)	71.0	41.1	41.12
Fat (%)	10.5	40.9	40.9
Protein (%)	18.5	18.0	18.0

^1^ CON, control diet with no added oil; SBO, metabolic syndrome diet + 13% soybean oil; LLO, metabolic syndrome diet + 13% low α-linolenic soybean oil.

^2^ In addition to the soy oil, other ingredients that supplied fat include corn, soybean meal, alfalfa meal and wheat middlings.

* Proximate composition of diets.

**Table 2:** Fatty acid composition of the different soy oils

**Fatty acid (%)**	**SBO**	**LLO**
Myristic acid	0.07	0.07
Palmitic acid	10.4	9.7
Stearic acid	4.1	4.6
Oleic acid	21.4	22.2
Linoleic acid	54.8	60.1
α-Linolenic acid	7.5	1.5
Arachidonic acid	ND	ND
Eicosapentaenoic acid	ND	ND
Docosahexaenoic acid	ND	ND
Omega 6 (n6)	54.8	60.1
Omega 3 (n3)	7.5	1.5
Omega 6: Omega 3 (n6/n3)	7.3	40.1

ND - not detected.

SBO, metabolic syndrome diet + 13% soybean oil; LLO, metabolic syndrome diet + 13% low α-linolenic soybean oil.

**Table 3:** Fatty acid composition of diets

**Fatty acid (%)**	**Treatment**^**1**^		
	**CON**	**SBO**	**LLO**
Myristic acid	0.30	0.08	0.10
Palmitic acid	14.08	10.93	10.55
Palmitoleic acid	0.30	0.10	0.13
Stearic acid	4.50	3.95	4.64
Oleic acid	21.95	19.71	21.27
Vaccenic acid	1.18	1.30	1.25
Linoleic acid	45.51	53.59	56.90
*cis9, trans11* CLA	ND^2^	0.23	0.09
*trans10, cis 12* CLA	ND	ND	ND
α-Linolenic acid	3.68	7.14	1.95
Arachidonic acid	ND	ND	ND
Eicosapentaenoic acid	ND	ND	ND
Docosahexaenoic acid	ND	ND	ND
Omega 6 (n6)	45.51	53.82	56.99
Omega 3 (n3)	3.68	7.14	1.95
Omega 6: Omega 3 (n6/n3)	12.4	7.5	29.2

^1^ CON, control diet with no added oil; SBO, metabolic syndrome diet + 13% soybean oil; LLO, metabolic syndrome diet + 13% low α-linolenic soybean oil.

^2^ ND - not detected.

### RNA isolation and cDNA synthesis

Isolated RNA was dissolved in nuclease free water (Ambion, Austin, TX) and concentrations were determined using a Nanodrop machine (Thermo Scientific, Waltham, MA). RNA samples were subjected to electrophoresis on a 1% agarose gel to check for RNA integrity. One microgram of total RNA was reverse transcribed with the MMLV reverse transcriptase (Promega, Madison, WI).

### Real-time PCR analysis

Real-time quantitative PCR was performed on a MyiQ real-time PCR detection system (Bio-Rad, Hercules, CA) using the SYBR RT-PCR mix (SABiosciences, Frederic, MD). The relative abundance of mRNA of the different genes was determined from the threshold cycle (Ct) for the respective genes after normalization with 18S which served as the internal control [12]. Primers used for RT-PCR are listed in Table 4.

**Table 4:** List of primers

**Gene**	**Forward**	**Reverse**
*18S*	5^′^-ATC CCT GAG AAG TTC CAG CA-3^′^	5^′^-CCT CCT GGT GAG GTC GAT GT-3^′^
*TNFα*	5'-CCA CCA ACG TTT TCC TCA CT-3^′^	5^′^-CCC AGG TAG ATG GGT TCG TA-3^′^
*IL6*	5^′^-TTC ACC TCT CCG GAC AAA AC-3^′^	5^′^-TCT GCC AGT ACC TCC TTG CT-3^′^
*Adiponectin*	5^′^-TGG AGA AAG CGC CTA TGT CT-3^′^	5^′^-TTT GCC AGT GGT GAC ATC AT-3^′^
*COL1A*	5^′^-GAC CGA GAC GTG TGG AAA C-3^′^	5^′^-CGC TGG GAC AGT TCT TGA TT-3^′^
*COLVIA*	5^′^-CGA CAT TGT GTT CCT GTT GG-3^′^	5^′^-TTC GTA AAC CGT GTC CAC AA-3^′^
*Fibronectin*	5^′^-AGC TGG AGG ACC AAG ACT GA-3^′^	5^′^-TGC CAT GAT ACC AAC AAG GA-3^′^
*CD68*	5^′^-ACG TTG GCT GTG CTC TTC TT-3^′^	5^′^-CTG GTG GTG GTA GCA GGA TT-3^′^
*MCP1*	5^′^-CAC CAG CAG CAA GTG TCC TA-3^′^	5^′^-TCC AGG TGG CTT ATG GAG TC-3^′^

### Western blot analysis

Adipose tissue was homogenized in RIPA lysis buffer (50 mmol/l Trizma-HCl (pH 7.4), 15 mmol/l NaCl, 0.25% deoxycholic acid, 0.1% Triton X, 10 mmol/l EDTA, 1 mmol/l Na_2_VO_3_ and protease inhibitor cocktail (Sigma-Aldrich). Homogenates were centrifuged at 10,000 × g for 10 min at 4°C to remove fat debris. Protein concentrations in homogenates were determined with the bicinchoninic acid (BCA) protein assay kit (Thermo Scientific, Waltham, MA). Protein samples were resolved on 10% SDS-PAGE gel and were transferred to a 0.2 μm nitrocellulose membrane using the semi-dry method (Bio-Rad, Hercules, CA). Membranes were blocked in 5% bovine serum albumin (BSA) in TBS (50 mmol/L Tris.HCl, pH 7.4, 150 mmol/L NaCl, 0.1% Tween 20). Immunoblotting for adiponectin was performed overnight at 4°C using a rabbit anti-porcine adiponectin antibody (kindly provided by Xeno Diagnostics, Indianapolis, IN) at a dilution of 1:1000. Membranes were stripped and reprobed with a rabbit anti-β-actin antibody (Cell Signaling Technologies, Danvers, MA) at a dilution of 1:1000. Blots were then incubated with a goat anti-rabbit secondary antibody conjugated to horseradish peroxidase (Cell Signaling Technologies, Danvers, MA) at a dilution of 1:20,000. Chemiluminescent signals from membranes were captured by autoradiography using the Immobilon (Millipore, Billerica, MA) chemilumniscent reagent. Densitometric analysis of western blots was performed using Kodak EDAS290 imaging system (Kodak, New Haven, CT).

### Determination of serum metabolites

Blood was collected from animals via the jugular vein after an overnight fast (8–10 hours). Whole blood was centrifuged at 4°C at 1500 × g for 15 minutes for collection of serum. Serum blood glucose concentration was determined with an automatic glucometer (Freestyle, Alameda, CA). Serum free fatty acid was determined using the free fatty acids half micro test kit (Roche Diagnostics, Indianapolis, IN). Serum triglyceride was determined with the triglyceride determination kit (Sigma Aldrich, St Louis, MO). Serum cholesterol concentration was determined using the Amplex Red cholesterol assay reagent (Invitrogen, Carlsbad, CA). High density lipoprotein (HDL) was separated for analysis from serum using an HDL kit (Pointe Scientific Inc., Canton, MI). All assays were performed according to instructions from the manufacturers.

### Insulin and C-reactive protein ELISA

Determination of serum insulin was conducted using the Mercodia porcine insulin ELISA kit (Mercodia, Uppsala, Sweden) and C-reactive protein (CRP) was determined using CRP kit (Immunology Consultants lab, Portland, OR) according to the manufacturer’s instructions.

### Tissue and serum fatty acids analysis

The modified procedure of Folch et al. [13] was used for total lipids extraction. Subcutaneous fat and serum samples were extracted in a 2:1 (vol/vol) chloroform: methanol organic solvent mixture [13]. Extracted fatty acids were dissolved in hexane for gas chromatographic analysis.

### Gas chromatography

Fatty acid methyl esters were quantified on a gas chromatography system (Varian 3900), using a CP wax 52 CB capillary column (Varian Inc., Palo Alto, CA). A Supelco PUFA-2 Component FAME Mix was used as the standard (Sigma-Aldrich, St. Louis, MO). Chromatographic profiles were evaluated for main fatty acid peak strengths.

### Statistical analysis

Data were examined for normality and analyzed using the GLM procedure (SAS Inst. Inc., Cary, NC). One-way analysis of variance model was used to test the data. When there was a significant main effect, separation of means was accomplished with the Tukey mean separation procedure. Differences were considered significant at *P* < 0.05 and at P < 0.10 for tendency towards significance. Values in texts represent means ± SEM.

## Results

### Fatty acid composition and animal performance

The fatty acid composition of experimental oils is presented in Table 2. The SBO has a linoleic acid and α-linolenic acid content of 54.8 and 7.5% respectively. The LLO has a content of 60.1 and 1.5% linoleic acid and α-linloenic acids respectively. The fatty acid composition of experimental diets is presented in Table 3. The CON, SBO and LLO diets have a linoleic acid content of 45.51, 53.59 and 56.90% respectively. The content of α-linolenic in the diets was 3.68, 7.14 and 1.95% for the CON, SBO and LLO diets respectively. The daily feed intakes across treatments were 1.55, 1.19 and 1.16 kg/day for the CON, SBO and LLO diets, respectively, and these were not different statistically. Estimated mean daily energy intakes were 4697.8, 5533.5 and 5394.0 kcal/day for the CON, SBO and LLO diets, respectively. However, because of the higher proportion of fat in the high fat diets (Table 1), estimated daily fat calories consumed in the diets were 512.1, 2263.2 and 2206.2 kcal/day for the CON, SBO and LLO diets, respectively. Total energy and fat calorie intake were similar in the SBO and LLO treatments, but higher than the CON. Although pigs were allowed to eat ad libitum, the total energy intake in the SBO and LLO diets was approximately 17% higher than in the CON diet. However, the fat calorie intake was 336% higher in the SBO and LLO diets than the CON diet. As expected, the differences in fat calories and the quantities of individual fatty acids consumed indicate that these were the main determinants of responses obtained in the diets. The estimated daily intake of fatty acids is presented in Table 5. The intake of linoleic acid in the CON, SBO and LLO diets were 27.51, 125.62 and 130.03 g/day. The intake of α-linolenic acid was 2.23, 16.73 and 4.46 for the CON, SBO and LLO diets respectively. The n6:n3 fatty acid intake ratios were 12.36, 7.54 and 29.20 for the CON, SBO and LLO diets respectively. Final mean body weights were 38.3, 40.3 and 39.3 kg for the CON, SBO and LLO treatments, respectively, and these were not significantly different.

**Table 5:** Estimated daily fatty acid intake per pig

**Fatty acid (g/d)**	**Treatment**^**1**^		
	**CON**	**SBO**	**LLO**
Myristic acid	0.18	0.19	0.23
Palmitic acid	8.51	25.62	24.11
Palmitoleic acid	0.18	0.24	0.29
Stearic acid	2.72	9.25	10.60
Oleic acid	13.27	46.20	48.61
Vaccenic acid	0.71	3.05	2.85
Linoleic acid	27.51	125.62	130.03
*cis9, trans11* CLA	ND	0.53	0.20
*trans10, cis 12* CLA	ND	ND	ND
α-Linolenic acid	2.23	16.73	4.46
Arachidonic acid	ND	ND	ND
Eicosapentaenoic acid	ND	ND	ND
Docosahexaenoic acid	ND	ND	ND
Omega 6 (n6)	27.51	126.16	130.22
Omega 3 (n3)	2.23	16.73	4.46
Omega 6: Omega 3 (n6/n3)	12.36	7.54	29.20

^1^ CON, control diet with no added oil; SBO, metabolic syndrome diet + 13% soybean oil; LLO, metabolic syndrome diet + 13% low α-linolenic soybean oil.

^2^ ND - not detected.

### Serum metabolite profile

The serum concentration of selected metabolites is presented in Table 6. Although the SBO diet resulted in marginal increase in the serum glucose concentration relative to the CON diet, it resulted in a significant increase serum insulin concentration. The LLO diet had a lower concentration of both glucose and insulin than the CON and SBO diets (P < 0.05). However, the SBO and LLO groups had higher serum non-esterified fatty acids (NEFA) concentration than the CON group (P < 0.05). Serum triglyceride concentration was higher in the SBO group compared to LLO and CON groups (P < 0.05). Total and LDL cholesterol concentrations were higher in the SBO group than in the CON and SBO groups. However, serum C-reactive protein (CRP) concentration was lower in the SBO and LLO diets than CON diet (P < 0.05).

**Table 6:** Effect of diets on serum metabolites

**Variable**	**Treatment**^**1**^			
	**CON**	**SBO**	**LLO**	**SEM**
Free fatty acids (mmol/L)	0.17^c^	0.28^b^	0.32^a^	0.01
Glucose (mmol/L)	8.8^b^	9.7^a^	7.8^c^	0.7
Insulin (pmol/L)	6.9^b^	30.9^a^	1.7^c^	0.7
Glucose: Insulin	1.28^b^	0.31^c^	4.59^a^	1.22
Triglycerides (mmol/L)	0.5^c^	1.5^a^	1.0^b^	0.03
C-reactive protein (mg/L)	101.4^a^	45.8^b^	65.3^b^	8.2
Total Cholesterol (mmol/L)	1.2^b^	4.9^a^	1.1^b^	0.08
HDL Cholesterol (mmol/L)	0.31^a^	0.14^b^	0.25^ab^	0.03
LDL Cholesterol (mmol/L)	0.8^b^	4.7^a^	0.9^b^	0.05

^1^ Data are given as means and pooled SEM, *n* = 4 per treatment group unless noted otherwise. CON, control diet with no added oil; SBO, metabolic syndrome diet + 13% soybean oil; LLO, metabolic syndrome diet + 13% low α-linolenic soybean oil. ^a,b,c^Labeled means in a row without a common superscript letter, P < 0.05.

HDL, high density lipoprotein; LDL, low density lipoprotein.

### Serum and tissue fatty acid profile

Serum and adipose tissue fatty acid composition are presented in Tables 7 and 8 respectively. The content of SFA was higher in the serum from CON group that the SBO and LLO groups (P < 0.05) (Table 7). Furthermore, the SBO group had a higher content of mono unsaturated fatty acids (MUFA), but a lower content of polyunsaturated fatty acids (PUFA) in the serum (Table 7) and subcutaneous fat tissue (Table 8), than the CON and LLO groups. Pigs in the SBO group had a higher MUFA: SFA ratio, but PUFA: SFA ratio was higher in the serum (Table 7) and subcutaneous fat tissue (Table 8) of pigs on the LLO diet. Additionally, pigs fed the SBO diet had a higher serum and subcutaneous fat tissue α-linolenic and oleic acid content. Serum and subcutaneous fat tissue linoleic acid content was also higher in the LLO group compared to the CON and SBO groups. However, stearic acid content was higher in both the serum and subcutaneous fat tissue of pigs on the CON diet than in those on the SBO and LLO diets. *Trans*10, *cis*12 CLA content was higher in the subcutaneous fat tissue of pigs in the CON group than in those in the SBO and LLO groups. However, *cis* 9, *trans*11 CLA content was higher in the subcutaneous fat tissue of pigs in the LLO group compared to those in the CON and SBO groups.

**Table 7:** Effect of diets on serum fatty acid profile

**Fatty acid (%)**	**Treatment**^**1**^			
	**CON**	**SBO**	**LLO**	**SEM**
Myristic acid	1.1^b^	3.5^a^	1.0^c^	0.01
Palmitic acid	23.1^a^	20.2^b^	19.3^c^	0.07
Palmitoleic acid	1.25^c^	1.60^a^	1.18^b^	0.01
Stearic acid	15.8^a^	12.0^b^	11.9^c^	0.03
Oleic acid	35.4^b^	38.8^a^	31.5^c^	0.06
Vaccenic acid	1.9^c^	2.7^a^	1.9^b^	0.01
Linoleic acid	14.3^b^	11.8^c^	25.8^a^	0.02
*cis9, trans11* CLA	1.0^b^	0.9^c^	1.2^a^	0.02
*trans10, cis 12* CLA	0.04^ab^	0.05^a^	0.04^b^	0.004
α-Linolenic acid	0.1^b^	0.8^a^	0.1^b^	0.01
Arachidonic acid	1.0^b^	0.6^c^	1.3^a^	0.01
Eicosapentaenoic acid	0.24^a^	0.16^c^	0.22^b^	0.01
Docosahexaenoic acid	0.16^a^	0.13^b^	0.15^ab^	0.01
Omega 6 (n6)	15.5^b^	12.6^c^	27.2^a^	0.02
Omega 3 (n3)	0.8^b^	1.4^a^	0.7^c^	0.02
Omega 6: Omega3 (n6/n3)	19.8^b^	9.3^c^	39.1^a^	0.7
Total SFA^2^	40.0^a^	35.6^b^	32.1^c^	0.07
Total MUFA^3^	39.6^b^	44.1^a^	35.5^c^	0.06
Total PUFA^4^	17.7^b^	15.2^c^	29.6^a^	0.03
MUFA: SFA	1.02^c^	1.24^a^	1.11^b^	0.003
PUFA: SFA	0.44^b^	0.43^c^	0.94^a^	0.001

^1^ Data are given as means and pooled SEM, *n* = 4 per treatment group unless noted otherwise. CON, control diet with no added oil; SBO, metabolic syndrome diet + 13% soybean oil; LLO, metabolic syndrome diet + 13% low α-linolenic soybean oil. ^a,b,c^Labeled means in a row without a common superscript letter, P < 0.05.

^2^ SFA- saturated fatty acid.

^3^ MUFA - Mono unsaturated fatty acid.

^4^ PUFA- Poly unsaturated fatty acid.

**Table 8:** Effect of diets on subcutaneous fat fatty acid profile

**Fatty acid (%)**	**Treatment**^**1**^			
	**CON**	**SBO**	**LLO**	**SEM**
Myristic acid	1.1^b^	3.3^a^	0.8^c^	0.02
Palmitic acid	23.3^a^	20.2^b^	18.3^c^	0.05
Palmitoleic acid	1.3^b^	1.6^a^	1.0^c^	0.004
Stearic acid	15.5^a^	12.5^b^	12.5^b^	0.04
Oleic acid	36.4^b^	39.2^a^	32.1^c^	0.12
Vaccenic acid	2.0^b^	2.8^a^	1.8^c^	0.01
Linoleic acid	14.2^b^	11.6^c^	27.5^a^	0.05
*cis9, trans11* CLA	1.0^b^	0.8^c^	1.2^a^	0.01
*trans10, cis 12* CLA	0.34^a^	0.26^c^	0.28^b^	0.003
α-Linolenic acid	0.05^c^	0.80^a^	0.09^b^	0.004
Arachidonic acid	1.0^b^	0.6^c^	1.4^a^	0.01
Eicosapentaenoic acid	0.23^a^	0.15^c^	0.22^b^	0.001
Docosahexaenoic acid	0.14^a^	0.10^b^	0.14^a^	0.004
Omega 6 (n6)	15.3^b^	12.4^c^	28.9^a^	0.04
Omega 3 (n3)	0.42^c^	1.05^a^	0.45^b^	0.004
Omega 6: Omega 3 (n6/n3)	37.2^b^	11.8^c^	65.3^a^	0.26
Total SFA^2^	40.1^a^	36.0^b^	31.7^c^	0.06
Total MUFA^3^	41.0^b^	44.6^a^	35.9^c^	0.13
Total PUFA^4^	17.5^b^	14.8^c^	31.3^a^	0.043
MUFA: SFA	1.03^c^	1.24^a^	1.16^b^	0.003
PUFA: SFA	0.44^b^	0.41^c^	1.06^a^	0.001

^1^ Data are given as means and pooled SEM, *n* = 4 per treatment group unless noted otherwise. CON, control diet with no added oil; SBO, metabolic syndrome diet + 13% soybean oil; LLO, metabolic syndrome diet + 13% low α-linolenic soybean oil. ^a,b,c^Labeled means in a row without a common superscript letter, P < 0.05.

^2^ SFA- saturated fatty acid.

^3^ MUFA - Mono unsaturated fatty acid.

^4^ PUFA- Poly unsaturated fatty acid.

### Inflammatory and extracellular matrix gene expression in adipose tissue

Expression of extracellular matrix and inflammatory genes was also determined. The expression of Col1A was lower in SBO group compared to LLO and CON groups (P < 0.05). There was also a tendency (P < 0.1) for a lower expression of COLVIA and fibronectin in the SBO treatment than the CON and LLO treatments (Table 9). However, expression of two inflammatory markers, MCP-1 and CD68 was not different in the subcutaneous tissue in the different dietary groups (P > 0.05) (Table 9). To determine the association fatty acid profile in the serum and adipose tissue and serum metabolite profile, a correlation analysis was conducted between these variables. Serum CRP was the only serum variable that was negatively correlated only to MUFA: SFA ratio (r = −0.53; P < 0.07). Correlation to individual fatty acids was weak (P > 0.1). The MUFA: SFA ratio was higher in both SBO and LLO diets and these diets had lower serum CRP concentration (Table 6). This suggests that the overall fatty acid profile and the sum of action of individual fatty acids may be very important in determining the metabolic response to the diets.

**Table 9:** Expression of inflammatory and extracellular matrix genes in subcutaneous adipose tissue

**Genes**	**Treatment**^**1**^			**SEM**	**P value**
	**CON**	**SBO**	**LLO**		
TNFα	0.870^b^	1.073^ab^	1.667^a^	0.229	0.05
IL-6	0.571^b^	1.705^a^	1.350^a^	0.212	0.05
Adiponectin	0.541^b^	1.055^a^	0.914^ab^	0.14	0.05
Col1A	1.681^a^	0.482^b^	1.733^a^	0.225	0.05
ColVIA	1.243	0.760	1.438	0.237	0.10
Fibronectin	1.363	0.701	1.390	0.232	0.10
CD68	1.243	0.885	1.373	0.254	0.10
MCP-1	0.872	1.256	1.247	0.198	0.10

^1^ Data are given as means and pooled SEM, *n* = 4 per treatment group. CON, control diet with no added oil; SBO, metabolic syndrome diet + 13% soybean oil; LLO, metabolic syndrome diet + 13% low α-linolenic soybean oil. ^a,b^Labeled means in a row without a common superscript letter differ, P < 0.05.

### Adiponectin expression

**Figure 1:**
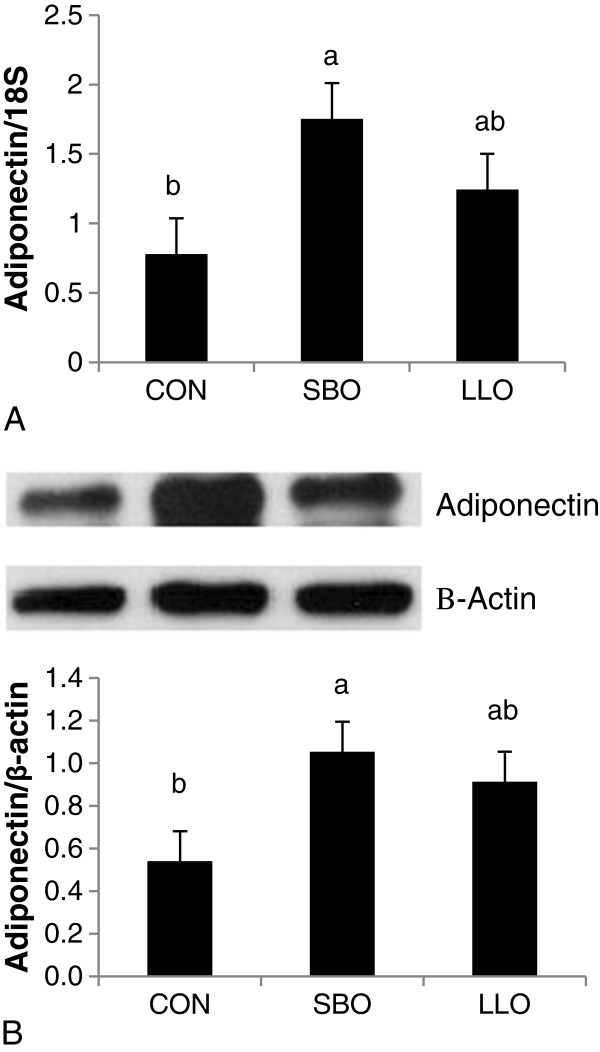
**Adiponectin gene expression in subcutaneous fat depot.** Expression of adiponectin in the subcutaneous adipose tissue by RT-PCR (**A**) or western blot (**B**). Bars represent mean ± SEM. Superscript letters represent significant mean differences (P < 0.05); n = 4.

Western blot analysis of adiponectin protein in the subcutaneous adipose tissue is presented in Figure 1. Higher expression of adiponectin protein was observed in pigs on the SBO diet compared to those on the CON diet (P < 0.05). However, similar levels were observed between the SBO and LLO groups.

## Discussion

Several advantages are associated with the use of the pig as a model for human nutrition research. These include its similar organ sizes compared to humans [14], similar digestive tract architecture, and similar lipid and carbohydrate metabolism [15-17]. Additionally, the Ossabaw pig is an excellent model for the study of metabolic syndrome as this animal model easily develops dyslipidemia when fed a diet high in fatty acids and cholesterol [18,19]. Consumption of a high fat, high calorie diet is also associated with increased risk of obesity, diabetes and cardiovascular disease [1,3]. Soy oil is a major vegetable oil consumed in the US [6]. It is rich in PUFAs which are highly susceptible to oxidative damage. Therefore, soy oil is usually hydrogenated to increase the shelf life of products made from it, a process that results in generation of *trans* fatty acids.

Consumption of high amounts of *trans* fat is linked to increased risk of cardiovascular diseases [20]. Lowering the content of α-linolenic acid to reduce soy oil unsaturation is a strategy for eliminating the need for soy oil hydrogenation and improving the shelf life of food products containing soy oil. Although this approach lowers the degree of unsaturation of soy oil, it increases its (n-6)/(n-3) ratio and the implications of long-term consumption of this oil on metabolic status are unknown. There has been no systematic study to characterize the metabolic response to consumption of the low α-linolenic compared to the standard soy oil. We report herein a comparison of several metabolic responses that may be associated with human consumption of the two types of soy oil using the pig model.

The estimated daily intakes of linoleic and α-linolenic acid in all treatments groups far exceeded the estimated daily intake for humans which ranged from 10.4-14.7 g/d for linoleic acid and 1.1-1.6 g/d for α-linolenic acid [21]. A major reason for this difference is the much larger feed intake in pigs relative to humans and the difference in feed composition between pigs and humans in general. The pig diet is also different from typical human diet due the absence of long chain PUFA such as eicosapentaenoic acid (EPA) and docosahexaenoic acid (DHA). The n6:n3 ratios of 12.36 and 7.54 in the control and SBO diets are not too far off from the 10.6:1 ratio in human diets [22]. However the ratio of 29.2 consumed in the LLO diet is quite different from human diet and far from the recommended n6:n3 of 4.0 in human diets [23]. The higher dietary n6:n3 ratio in the LLO diets is further reflected in the elevation of this ratio in the serum and subcutaneous adipose tissue as well. This underscores the disruption that may occur to this ratio in serum and adipose tissue if low α-linolenic acid represents a major source of n3 fatty acids in human diets. Because insulin resistant state if often marked by elevated serum insulin and glucose concentrations [24,25], the elevated glucose and insulin concentrations in the SBO diet relative to the CON and LLO diets could suggest development of insulin resistance in pigs on this treatment, and indicate that the LLO oil prevents pigs from developing insulin resistance, despite consuming similar level of dietary fat as in the SBO diet. Likewise, the higher levels of triglycerides, LDL-cholesterol and the lower level of HDL-cholesterol in pigs on the SBO diet may point to development of dyslipidemia, a condition that is marked by elevated levels of blood lipids [26]. Thus consumption of LLO oil may offer some protection against development of both insulin resistance and dyslipidemia. Therefore, the disruption of the n6:n3 ratio in the LLO did not result in a significant adverse metabolic response. Consumption of both SBO and LLO diets resulted in a lower level of CRP than in pigs on the CON diet. CRP is a cardiovascular disease marker [27]. The reduction in CRP concentration in the SBO and LLO diets is consistent with the reported effect of PUFA in reducing CRP concentration [28,29] and indicates that despite the reduced content of α-linolenic acid, the low α- linolenic oil was effective in lowering the concentration of this cardiovascular disease marker and this might indicate that α-linolenic acid may not be the major factor in soy oil regulating the concentration of CRP. Soy is rich in linoleic acid, and due to its anti-inflammatory property, this fatty acid could be the major player in lowering the concentration of CRP [28,29].

Several distinct differences in the serum fatty acid profiles between pigs on the SBO and LLO diets could explain the different serum metabolic profile in the pigs. The LLO diet resulted in higher *cis* 9, *trans*11 CLA, reduced total SFA, increased total PUFA content and higher PUFA: SFA ratio in the serum than the SBO diet. It has been reported previously that dietary inclusion of *cis* 9, *trans*11 CLA in mice led to improved glucose tolerance, insulin sensitivity and reduction in triacylglycerol content compared to control fed mice [30,31]. Choi et al. [32] showed a higher response to insulin in rats whose diets were supplemented with CLA. Furthermore, inclusion of *cis* 9, *trans*11 CLA in ob/ob mice improved insulin signaling [31]. SFAs have been shown to impair insulin signaling [33] whereas PUFAs enhance it [34]. Therefore, the overall changes in the fatty acid profile in the LLO treatment may support enhanced insulin sensitivity *in vivo*. Increased insulin sensitivity will also result in reduced serum cholesterol concentration [35]. This is in agreement with the reduced serum cholesterol content in the serum of LLO pigs observed in this study. As found earlier [36], consumption of *cis* 9, *trans*11 CLA by ApoE knockout mice resulted in reduced triglycerides, improved glucose tolerance and insulin sensitivity compared to control fed mice. Thus *cis* 9, *trans*11 CLA may have played a big role in the apparent enhanced insulin sensitivity in the LLO diet. Finally, the lower SFA, higher PUFA content and higher PUFA: SFA ratio in the LLO fed pigs could have contributed to their improved metabolic profile as well. Consumption of lower amounts of SFA and consumption of higher amounts of PUFA are both associated with reduction in risks for development of inflammation and metabolic syndrome [37-39]. Although associations between consumption of individual fatty acids and serum metabolites are well established [28-39], it is probable that the overall fatty acid profile and the complex interactions it engenders between individual components may play important roles in determining the dietary effects observed. A comprehensive metabolomics analysis may be needed in the future for such a determination. Obesity is currently regarded as low-grade chronic inflammatory disease [40] and consumption of n3 fatty acid enriched diets may offer protection against obesity-induced inflammation [41]. In this study consumption of both SBO and LLO diets resulted in elevated adipose tissue expression of IL-6. However, the higher expression of TNFα in the adipose tissue of pigs on the LLO diet than those on the CON diet may suggest a potential loss of some anti-inflammatory effects of ALA in the LLO diet and this could be a disadvantage of consuming a diet low in ALA. Nevertheless, the overall limited effect of reducing ALA level in the diet of pigs on the LLO diet may suggest a weak link between ALA and the regulation of inflammation. Indeed, in healthy human subjects with large waist circumferences, increased ALA consumption from flaxseed oil consumption failed to reduce inflammatory makers [42]. Therefore, the importance of ALA consumption in regulating adipose tissue inflammation could be complex and additional studies are required to further elucidate the importance of ALA in the regulation of inflammation.

Adiponectin is an adipocyte-derived hormone that can improve insulin sensitivity by regulating glucose utilization and fatty acid metabolism. It is possible that the elevated serum and subcutaneous adipose tissue adiponectin abundance in the SBO group is related to the elevated oleic acid abundance in the serum and subcutaneous adipose tissue of pigs in this group. In support of this hypothesis, a recent study by Granados et al. [43] showed that incubation of 3T3-L1 adipocytes with oleic acid increased adiponectin mRNA levels. Positive association between adipose tissue oleic acid content and serum adiponectin concentration has also been reported [44]. Therefore, elevated oleic acid level in the serum and adipose tissue of SBO pigs may partly contribute to the higher expression of adiponectin in this treatment.

In summary, we report herein that feeding the low α-linolenic soy oil resulted in alteration of serum metabolite profile marked by reduced serum glucose, insulin, triglycerides and total and LDL cholesterol concentrations compared to regular soy oil. However, there were no significant changes in the expression of inflammatory markers. We speculate that these changes may be driven by the reduction in SFA content, elevation of PUFA and *cis* 9, *trans*11 CLA content and the increase in PUFA: SFA ratio following consumption of the LLO diet.

## Competing interests

The authors declare no conflict of interest.

## Authors’ contributions

RBP, OA and KMA designed the research. RBP, HL and KMA conducted the research. RBP and KMA analyzed the data. RBP, HL and KMA wrote the paper. KMA had primary responsibility for the final content. All authors read and approved the final manuscript.
